# A risk score model for the prediction of osteosarcoma metastasis

**DOI:** 10.1002/2211-5463.12592

**Published:** 2019-02-02

**Authors:** Siqi Dong, Hongjun Huo, Yu Mao, Xin Li, Lixin Dong

**Affiliations:** ^1^ Surgeon of Orthopedics Department II First Hospital of Qin Huangdao China; ^2^ Baotou Medical College China; ^3^ Department of Oncology First Hospital of Qinhuangdao China

**Keywords:** metastasis: bioinformatic analysis, osteosarcoma, risk score model

## Abstract

Osteosarcoma is the most common primary solid malignancy of the bone, and its high mortality usually correlates with early metastasis. In this study, we developed a risk score model to help predict metastasis at the time of diagnosis. We downloaded and mined four expression profile datasets associated with osteosarcoma metastasis from the Gene Expression Omnibus. After data normalization, we performed LASSO logistic regression analysis together with 10‐fold cross validation using the GSE21257 dataset. A combination of eight genes (*RAB1*,*CLEC3B*,*FCGBP*,*RNASE3*,*MDL1*,*ALOX5AP*,*VMO1* and *ALPK3*) were identified as being associated with osteosarcoma metastasis. These genes were put into a gene risk score model, and the prediction efficiency of the model was then validated using three independent datasets (GSE33383, GSE66673, and GSE49003) by plotting receiver operating characteristic curves. The expression levels of the eight genes in all datasets were shown as heatmaps, and gene ontology gene annotation and Kyoto Encyclopedia of Genes and Genomes pathway enrichment analysis were performed. These eight genes play a role in cancer‐related biological processes, such as apoptosis and biosynthetic processes. Our results may aid in elucidating the possible mechanisms of osteosarcoma metastasis, and may help to facilitate the individual management of patients with osteosarcoma after treatment.

**Abbreviations**
AUC: area under receiver operating characteristic curve
GEO: Gene Expression Omnibus
GO: gene ontology
KEGG: Kyoto Encyclopedia of Genes and Genomes
LASSO: least absolute shrinkage and selection operator
ROC: receiver operating characteristic

As the most common primary malignant bone tumor in childhood and adolescence, osteosarcoma exhibits highly aggressive and early systemic metastasis 1, 2. Osteosarcoma systemic metastasis, especially pulmonary metastasis, is still the most prominent reason for osteosarcoma‐caused death as over 90% of patients with osteosarcoma die from pulmonary metastases 3, 4. Despite great advancement in the treatment for osteosarcoma, only 11–30% of patients suffering from osteosarcoma metastasis survive after the combination of surgery resection and chemotherapy 5, 6. Hence, it is of great importance to explore novel biomarkers and therapeutic targets for osteosarcoma metastasis prediction. In recent years, developments in molecular biology have provided new insights into potential diagnostic and therapeutic biomarkers for osteosarcoma. Previous study demonstrated that prometastasis genes such as MYC 7, 8 and RAS 9 facilitate osteosarcoma metastasis and metastasis‐resistant genes including nm23 10, p16 11 and KiSS‐1 metastasis‐suppressor 12 inhibited the metastasis process in osteosarcoma. Furthermore, microarray technology has been widely used for screening a series of metastasis‐related genes in osteosarcoma 13, 14.

On the other hand, recent release of gene expression microarray profile data and clinical information in the Gene Expression Omnibus (GEO) and The Cancer Genome Atlas has provided large amounts of microarray data that can be applied to identify a series of highly specific and sensitive markers. Gene expression profiling based on these datasets has been utilized to identify critical genes associated with metastasis 15, 16. For example, differentially expressed pathways related to the metastasis of osteosarcoma were identified by performing bioinformatics analysis based on GEO data 14. A series of osteosarcoma metastasis‐associated genes was also identified by performing weighted gene coexpression network analysis 13. Besides, the gene expression signature has aroused great attention and has been widely constructed to predict the metastasis and prognosis of different cancers.

In order to help predict the metastasis at time of diagnosis, we downloaded and mined four gene expression microarray datasets from GEO which were used as a training set or validation set. After normalization, we performed the least absolute shrinkage and selection operator (LASSO) logistic regression model along with 10‐fold cross validation to construct a metastasis prediction score model. Receiver operating characteristic (ROC) curves were plotted to validate the prediction efficiency of the model. Finally, metastasis‐associated genes were put into gene ontology (GO) biological process enrichment and Kyoto Encyclopedia of Genes and Genomes (KEGG) signaling pathways analysis.

## Materials and methods

### Gene expression profiles and data pre‐processing

Gene expression datasets were retrieved from GEO using the key words ‘osteosarcoma’ and ‘metastasis’. Four datasets that met the following criteria were downloaded: gene expression data and information about metastasis were described. Four gene expression datasets, namely GSE21257 (total number: 53, metastases: 14), GSE33383 (total number: 53, metastases: 34), GSE66673 (total number: 24, metastases: 12) and GSE49003 (total number: 12, metastases: 6), were retrieved with Affymetrix platforms. The metastasis information and samples used for microarray analysis of these patients were collected at the time of diagnosis. Then background correction and normalization were performed using r software 17 and bioconductor 18. In order to reduce non‐biological variability across arrays, the gene expression profiles in different datasets were quantile normalized separately. The quantile normalization forces the distributions of the samples to be the same on the basis of the quantiles of the samples by replacing each point of a sample with the mean of the corresponding quantile 19, 20. Briefly, these normalization methods firstly arrange the logarithmic transformed microarray data into a *G* × *N* matrix *X*, where *G* and *N* are total numbers of genes and arrays, respectively; sort each column of *X* to give *X*_sort_; take the means across the rows of *X*_sort_ and assign this mean to each element in the row to get *X*_sort_; and finally obtain the normalized version *X*_norm_ of *X* by rearranging each column of *X*_sort_ to have the same ordering as in the original *X* 21. Subsequently, probes were mapped to gene symbols. Empty probes were discarded according to the annotation platform of each expression profile. Average expression values were calculated for duplicated samples and missing values were estimated using weighted K‐nearest neighbors 22.

### Construction of metastasis prediction of risk score model

A logistic regression model along with the LASSO method for variable selection and shrinkage was applied to build a metastasis prediction of risk model by using the r package glmnet (https://CRAN.R-project.org/package=glmnet) 23. The penalty regularization parameter λ was determined via the cross‐validation routine cv.glmnet before running the main algorithm with an *n*‐fold value equal to 10. The λ value was finalized by using lambda.min, which is the value of lambda giving minimum mean cross‐validated error 23, 24, 25. A series of genes combined with the corresponding efficiency were identified from the GSE21257 training set and used to construct a metastasis prediction of risk score model. Based on the model, the risk score for each individual was calculated.

### Validation of the risk score model

In order to confirm the robustness and accuracy of the risk score model, the remaining three datasets (GSE33383, GSE66673 and GSE49003) were used as validation sets. The classification effect was comprehensively evaluated in terms of area under the ROC curve (AUC).

### Function enrichment analysis

Genes from the risk score model were put into GO biological function and KEGG enrichment analysis to elucidate the biological implications of the genes in the signature. cytoscape software (National Institute of General Medical Sciences, Bethesda, MD, USA) combined with ClueGO and CluePedia Plugins was applied to perform the enrichment analysis.

## Results

### Data preprocessing and risk score model construction

**Figure 1:**
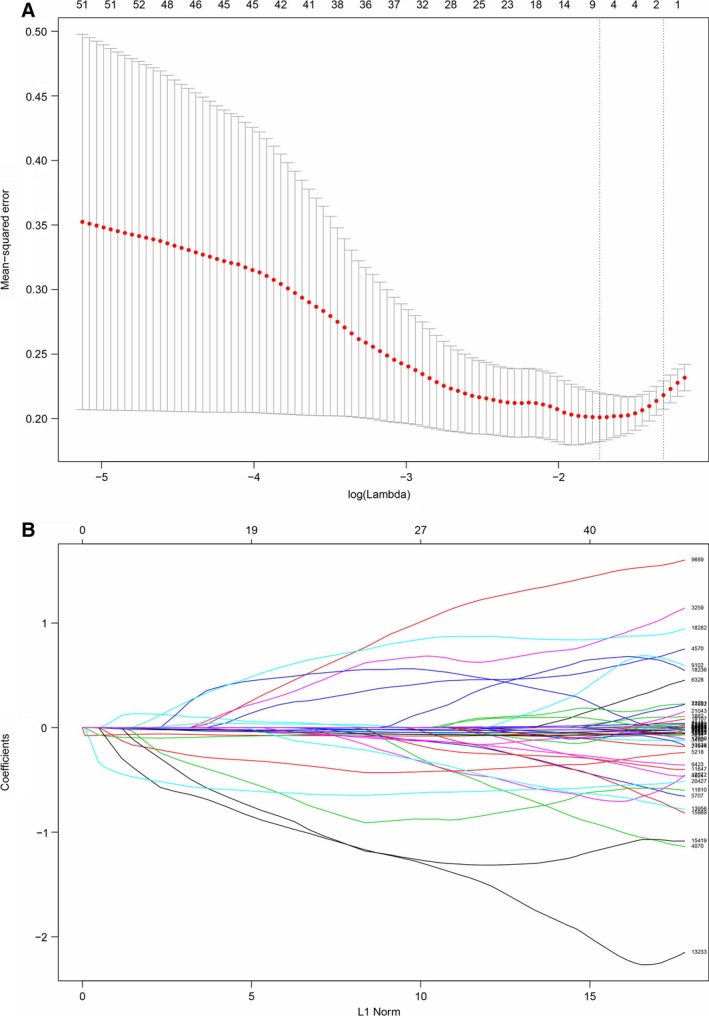
Risk score model construction using LASSO logistic regression analysis along with 10‐fold cross validation. (A) Partial likelihood deviance was plotted *versus* log(Lambda). The vertical dotted line indicates the lambda value with the minimum error and the largest lambda value where the deviance is within one SE of the minimum. (B) LASSO coefficient profiles of the genes associated with the metastasis of osteosarcoma.

Based on the expression profile of GSE21257, we used a LASSO logistic regression combined with 10‐fold cross validation to build a classifier to predict metastasis in patients with osteosarcoma (Fig. 1). A combination of eight genes was selected as the best predictor of metastasis in the training cohort: *RAB1*,* CLEC3B*,* FCGBP*,* RNASE3*,* MDL1*,* ALOX5AP*,* VMO1* and *ALPK3*. A risk score formula was derived to calculate a risk score of metastasis for each patient based on the expression level of eight genes: *RAB1* × −0.286 + *CLEC3B* × −0.073 + *FCGBP* × −0.061 + *RNASE3* × −0.548 + *MDL1* × −0.139 + *ALOX5AP* × −0.017 + *VMO1* × −0.002 + *ALPK3* × 0.092.

### Expression profile of key genes in different datasets

**Figure 2:**
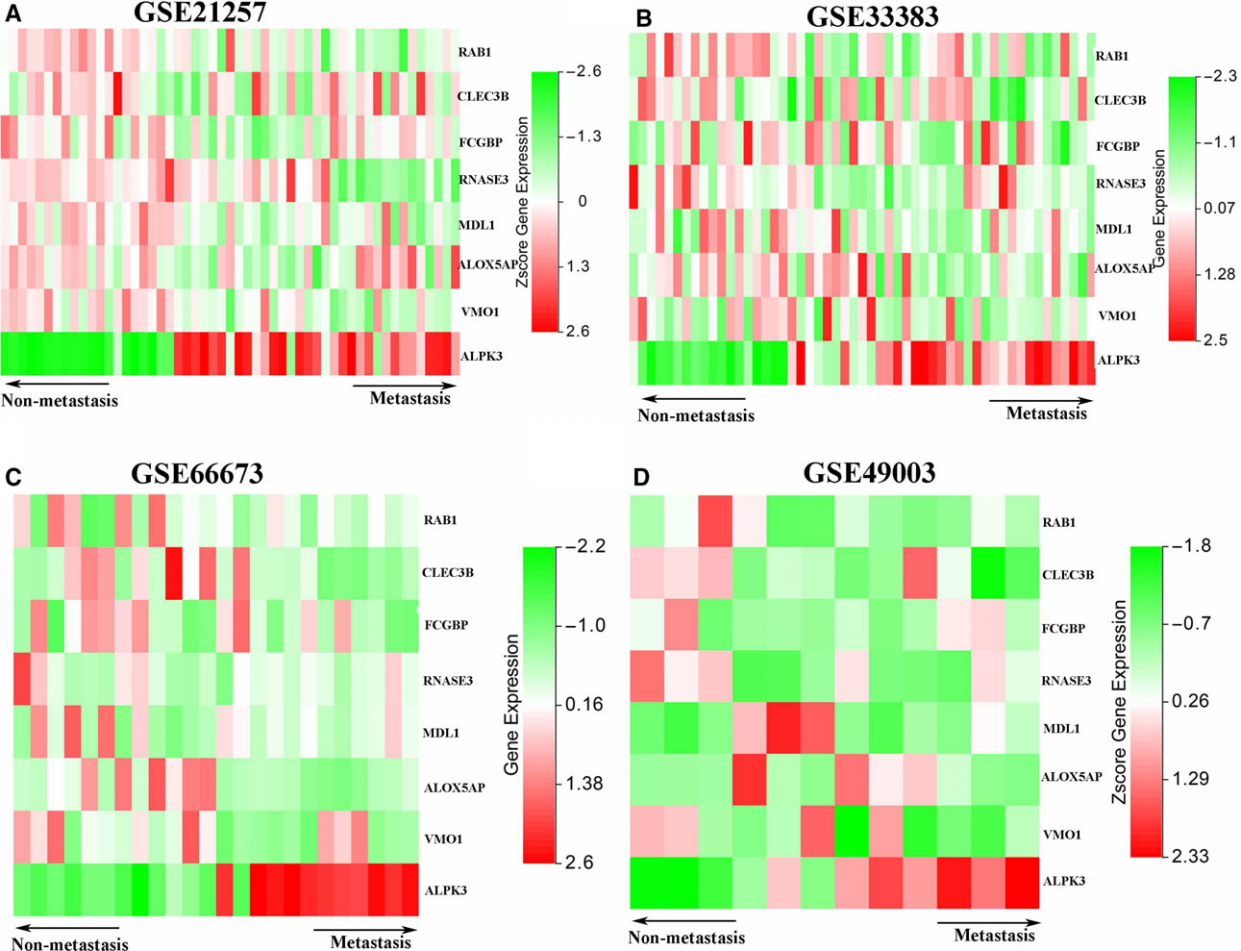
The expression level of eight genes in all the four datasets. Heatmaps were plotted to reveal the expression level of eight genes in GSE21257 (A), GSE33383 (B), GSE66673 (C) and GSE49003 (D) datesets.

The expression level of eight genes from the signature was plotted as a heatmap and shown in Fig. 2. According to the results, the expression levels of *RAB1*,* CLEC3B*,* FCGBP*,* RNASE3*,* MDL1*,* ALOX5AP* and *VMO1* were relatively lower in patients with metastatic osteosarcoma than that in non‐metastatic osteosarcoma. On the contrary, patients with metastatic osteosarcoma tended to have a higher expression level of *ALPK3* than those with non‐metastatic osteosarcoma. Similar results were observed not only in the training set (GSE21257) but also in the other three datasets (GSE33383, GSE66673 and GSE49003).

### Stability and validity verification

**Figure 3:**
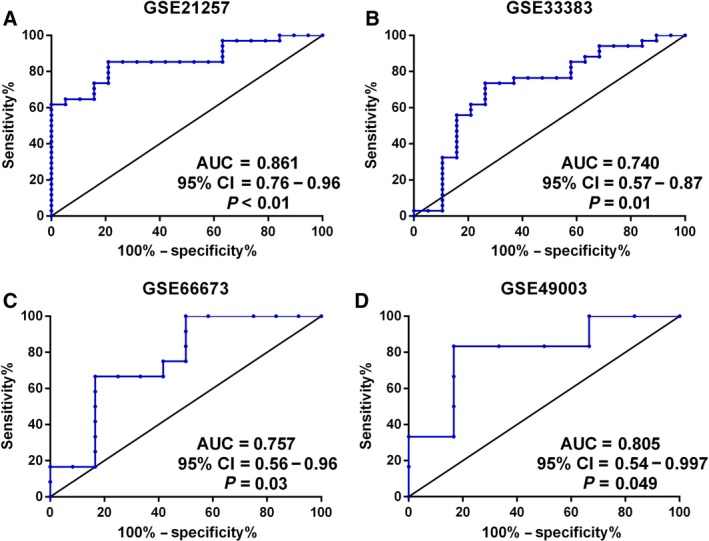
Prediction efficiency of the gene risk score was evaluated using ROC curves. The ROC curves are shown for risk score model in GSE21257 (A), GSE33383 (B), GSE66673 (C) and GSE49003 (D) datesets.

GSE21257, GSE33383, GSE66673 and GSE49003 datasets were all utilized to verify the robustness and transferability of the risk score model generated by the LASSO logistic regression. The ROC curves were plotted to assess the prediction accuracy. According to the results in Fig. 3, the risk score model can distinguish the metastatic individuals from the non‐metastatic individuals with high accuracy (AUC = 0.861, *P* < 0.01). Moreover, independent cohorts were also collected to act as an external validation cohort. High accuracy was also demonstrated in three independent cohorts, which suggested the stability of the risk score model.

### Functional enrichment analysis of genes from the risk score model

**Figure 4:**
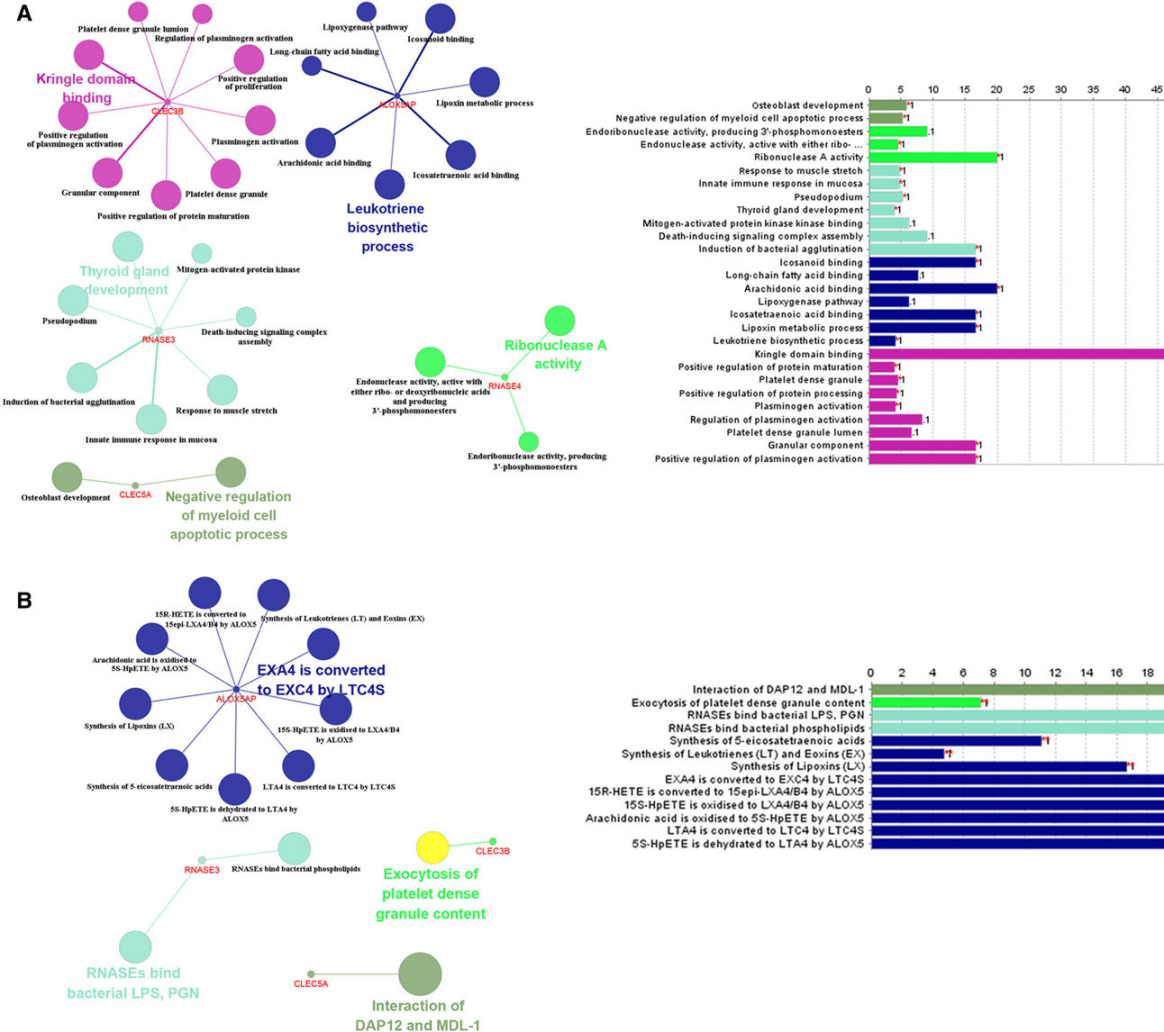
Functional enrichment analysis depicting the biological pathways and processes associated with genes in the risk score. The results are shown of GO biological process enrichment (A) and KEGG signaling pathways analysis (B).

In order to identify the biological pathways and processes correlated with the eight genes, GO biological process enrichment and KEGG signaling pathways analysis were performed. According to the results, the eight genes play important roles in cancer‐related biological processes such as cell apoptosis and the leukotriene biosynthetic process (Fig. 4).

## Discussion

Metastasis is the main factor that affects the prognosis of osteosarcoma, and several factors such as differential gene expression are involved in this progress. Early diagnosis or prediction of metastasis is rather critical considering there is a great difference in the survival rate between patients with metastatic osteosarcoma (10–20%) and non‐metastatic osteosarcoma (50–78%) 26, 27. Hence, construction of a prediction or early diagnosis model would benefit the treatment and prognosis evaluation.

In the present study, we downloaded and mined four gene datasets from GEO and further construct a risk score model. As the gene expression profiles were downloaded from four datasets, the data were firstly normalized. Normalization aims to make the samples of the data more comparable and the following downstream analysis reliable. After normalization, we fitted a logistic regression model and used LASSO for variable selection and shrinkage, which is a well‐established method for selection of the most predictive markers with high throughput data. The LASSO logistic regression model allows integration of multiple biomarkers into one tool providing more accurate prediction of disease progression than single biomarkers alone. The regularization parameter was chosen as the largest value where the error was within 1 standard error of the minimum as determined by 10‐fold cross validation 23, 25, 28. Considering the microarray expression profile used in the present study is of high‐throughput biological data, the common problem, ‘curse‐of‐dimensionality’ (small sample size combined with a very large number of genes) was taken into consideration. On the other hand, LASSO manages high‐dimensional regression variables with no prior feature selection step by shrinking all regression coefficients toward zero and thus forcing many regression variables to be exactly zero 29. Consequently, a series of variables along with the regression coefficients were selected and a formula was constructed to act as a risk score model for the prediction of osteosarcoma metastasis. Therefore, the LASSO model can be applied to solve the ‘curse‐of‐dimensionality’ problem.

To further elucidate the underlying mechanism of metastasis in osteosarcoma, genes in the risk score model were put into annotation and function enrichment analysis. These genes were found to be involved in several cancer‐related activities such as cell apoptosis and the leukotriene biosynthetic process. Previous studies have identified that *RAB1* plays a role in squamous carcinoma cervical cancer 30. *CLEC3B* is down‐regulated and inhibits proliferation in clear cell renal cell carcinoma 31. The participation of *FCGBP* in gastric tumorigenesis and progression was also revealed 32, and *FCGBP* is validated as a key regulator of the epithelial–mesenchymal transition process that contributed to the metastasis and prognosis of gallbladder cancer 33. Moreover, the expression levels of *ALOX5AP* are significantly correlated with the survival time of esophageal squamous cell carcinoma patients 34. Whether the influence of these genes may also have an effect in osteosarcoma and contribute to the progression of the osteosarcoma deserves further exploration.

Our study here identified some core genes in the metastasis of osteosarcoma and further constructed a risk score model, which may facilitate further exploration of mechanisms. However, there are some limitations to our study. First, the numbers of patients in all the four GEO datasets are relatively small. More patients and clinical information should be collected to further validate the stability of the model. Second, some genes might be excluded because of our rigorous screening criteria. Third, the function annotation analysis of target genes was based on bioinformatics analysis. More experiments will be needed for validation or even correction and to confirm the KEGG pathway analysis and GO enrichment results.

In conclusion, we constructed an eight‐gene risk score by performing logistic regression analysis along with 10‐fold cross validation based on datasets downloaded from GEO. The stability and accuracy were further assessed in three independent cohort. Future studies suggested that genes from the risk score participate in several cancer‐related biological processes. This risk score model has provided new insight into the prediction of osteosarcoma metastasis and has potential prognostic and therapeutic implications for osteosarcoma.

## Conflict of interest

The authors declare no conflict of interest.

## Author contributions

SD contributed to the study design, data profiling and manuscript draft. YM downloaded and analyzed data. HH, XL and LD performed language editing. Final manuscript was reviewed and approved by all the authors reviewed.
